# A landscape review to identify what matters to patients with thrombotic cardiovascular diseases and patient-reported outcome instruments which can be used to capture the patient experience

**DOI:** 10.1007/s11136-024-03790-1

**Published:** 2024-11-22

**Authors:** Alexandra I. Barsdorf, John Fastenau, Shannon Lee, Xiaoyan Li, Ellen O’Brien, Blue Stevenson, Brandon Becker

**Affiliations:** 1 Clinical Outcomes Solutions, Chicago, IL USA; 2 Janssen Global Services, LLC, a Johnson & Johnson Company, Raritan, NJ USA; 3 FingerPost Consulting, Cheshire, UK; 4 Bristol Myers Squibb, Lawrenceville, NJ USA; 5 Janssen Global Services, LLC, a Johnson & Johnson Company, Horsham, PA USA

**Keywords:** Coronary artery disease, Atrial fibrillation, Stroke, Patient-reported outcome, Literature review

## Abstract

**Purpose:**

Thrombotic cardiovascular diseases profoundly impact patients’ health-related quality of life (HRQoL). However, patient-reported outcome (PRO) instruments that are disease-specific or antithrombotic-treatment focused, developed according to US Food and Drug Administration (FDA) guidance on PROs, and can be used in clinical trials, are lacking. The aim of this study was to understand concepts important to patients diagnosed with coronary artery disease (CAD) or acute coronary syndrome (ACS), atrial fibrillation (AF), or stroke who require antithrombotic treatment for reducing risk of future thrombotic events (indications being evaluated for an investigational new drug), identify PROs that measure relevant symptoms and impacts, and determine acceptability of PROs from a health technology assessment (HTA) perspective.

**Methods:**

A landscape review, conducted between January 2009 and October 2020, included a search of qualitative literature (OVID), a review of PRO instruments using multiple sources (e.g., OVID and clinical trials databases), and a survey of HTA decisions for antithrombotic medications.

**Results:**

The qualitative literature review identified 27 publications used to develop a high-level conceptual summary of symptoms and HRQoL impacts reported by patients. The instrument landscape review indicated that generic PROs have been utilized for thrombotic indications, but disease-specific, fit-for-purpose instruments are lacking, and the HTA review revealed that although HTA agencies discussed PRO instruments, evidence of specific recommendations was not found.

**Conclusion:**

To ensure patients’ experiences, perspectives, and priorities are incorporated into drug development and evaluation, a core set of PROs for thrombotic indications that meet health authority guidance and are acceptable to HTA agencies is needed.

**Supplementary Information:**

The online version contains supplementary material available at 10.1007/s11136-024-03790-1.

## Introduction

Cardiovascular diseases (CVDs) are a leading cause of morbidity and mortality worldwide. According to the World Health Organization, in 2019, an estimated 17.9 million people died from CVDs. This represents 32% of all global deaths. Strategies to reduce CVD risk include patients’ adoption of healthy behaviors and guideline-directed medical care for hypertension, diabetes, and dyslipidemia [1]. The majority of CVD-related deaths are due to thrombotic conditions including acute coronary syndrome (ACS), coronary artery disease (CAD), and atherothrombotic cerebrovascular accidents, including stroke associated with and without atrial fibrillation (AF) [2]. Anticoagulant and antiplatelet drugs are frequently used to prevent clot formation in patients with CAD or ACS (a subset of CAD), AF, or stroke who require antithrombotic treatment for reducing risk of future thrombotic events.

Thrombotic indications have serious impacts on the lifestyle and health-related quality of life (HRQoL) of patients. Patients report physical limitations such as difficulties with mobility [3], sleep issues [4, 5], and challenges with everyday activities and work [6]. Patients also report social and emotional issues such as fear of death, anxiety, and stress [7, 8], and being a burden to their family members and friends. Thrombotic indications also require dietary and lifestyle modifications to reduce risk. Patients and physicians often have concerns about bleeding risk associated with anticoagulant and antiplatelet drugs [9, 10]. While several patient-reported outcome (PRO) instruments have been utilized to capture symptoms and impacts in patients with CAD/ACS, AF, or stroke, many of these were not developed specifically for use in these patient populations or are not fit-for-purpose measures developed according to the 2009 US Food and Drug Administration (FDA) PRO guidance [11]. It is important to capture the patient experience in drug development and ensure that PROs being used measure concepts relevant to patients’ experience of the condition. The importance of this is highlighted in the FDA’s patient-focused drug development (PFDD) initiative which was developed to obtain patients’ perspectives on specific diseases and treatments through FDA public meetings.

The objectives of this study were to (i) develop a high-level conceptual summary of relevant symptoms and impacts based on reports by patients across select thrombotic indications, (ii) identify PRO instruments used in either clinical research or practice to capture relevant symptoms and impacts, including those related to physical function and symptomatic treatment effects associated with anticoagulant and antiplatelet use, and determine their suitability for use as a core set, and (iii) understand how the evidence generated is currently used by regulatory and health technology assessment (HTA) stakeholders to inform decision-making.

A landscape review of HRQoL in patients with CAD/ACS, AF, or stroke and PRO instruments used in clinical research or practice was performed. These thrombotic indications were selected as a new investigational drug is being explored across these patient populations. The aim of the review was to search multiple data sources to understand what is important to measure from a patient’s perspective, identify PROs that measure relevant symptoms and impacts, and determine acceptability of PROs from a HTA perspective to support development of a core set of meaningful PROs. A core outcome set is defined as “an agreed standardized set of outcomes that should be measured and reported, as a minimum, in all clinical trials in specific areas of health or health care [12].” There is a drive to develop core outcome measures in clinical trials [13] and for PROs, such as for cancer [14] and inflammatory bowel disease [15]. In addition, a review of the HTA landscape was conducted to determine acceptability of PRO instruments from an HTA perspective. HTA helps to determine access, reimbursement, and pricing strategy for medical products. The HTA processes, evidence requirements, and reasons for evaluation differ across markets and HTA organization. It is valuable to consider which outcomes are most likely to meet most requirements by looking at previous appraisals for the same condition across multiple markets.

## Methods

### Qualitative literature review

A targeted search of the literature was conducted in OVID using Embase, MEDLINE, and PsycINFO to identify English language qualitative literature in the select thrombotic indications to inform the development of a high-level conceptual summary depicting core symptoms and impacts reported by patients. OVID was used as OVID Medline, which includes the same records as PubMed Medline, and allows for a more focused search to be performed within Medline publications. Select MESH and/or free text search terms were developed to facilitate the identification of the most relevant qualitative studies (Table 1). Date limitations were applied to restrict the search results as needed; searches were from January 2009 to October 2020 to cover the period for the implementation of the 2009 FDA PRO Guidance [11] and were limited to English language and Human studies. Exclusion of articles was based on objectives of the study and was further defined during the review of articles.

**Table 1:** OVID literature review search terms

OVID qualitative literature review	OVID PRO instrument review	
Qualitative research	Patient-reported outcome	Caregiver-reported outcome*
Interview	Quality of life	Instrument*
Semi structured interview	Health survey	Observer reported*
Structured interview	Questionnaire	Caregiver reported*
Telephone interview	Self report	Measure*
Unstructured interview	Rating scale	Evaluation*
Personal experience	Exp functional status assessment	Anticoagulant
Narrative	Functional assessment	Antiplatelet
Conceptual framework	Self evaluation	Blood thinners
Focus group*	Symptom assessment	Antithrombotic
Patient perspective*	Numeric rating scale	
Patient input*	Likert scale	
Patient opinion*	Visual analog scale	
Subjective experience*	Patient-reported outcome*	

Note: The asterisk wildcard character (*) broadens a search by including alternative forms of words such as plurals

### PRO instrument review

#### Review of PRO instruments

A landscape review was conducted to identify PRO instruments used with patients diagnosed with CAD/ACS, AF, or stroke who require antithrombotic treatment for reducing risk of future thrombotic events. This type of review allows for the search of multiple sources of data (e.g., literature, clinical trials, clinical guidelines, and integration of findings) to help inform decision-making. Suitability of potential instruments for inclusion in a core set was evaluated based on the totality of evidence (e.g., review of instrument domains for inclusion of concepts relevant to patients as depicted in the high-level conceptual summary) and review of instrument psychometric properties in the populations of interest.

English language studies in OVID published from January 2009 to September 2020 were searched using MESH and/or free text outcome terms (Table 1). Generic (designed to be used across various diseases/illnesses, populations, and interventions) and disease-specific (specific to a disease/illness) instruments used in either clinical practice or research were included to ensure a comprehensive search and to understand what is being used in these patient populations. PRO instruments used to assess HRQoL pre- and post-surgical intervention were excluded as these were not in scope for this study.

Clinical trial databases (ClinicalTrials.gov and ClinicalTrialsRegister.eu) were searched from 2009 to 2020 to identify studies that included relevant PRO instruments used as outcome measures. Each indication [CAD/ACS, secondary stroke prevention (SSP), and stroke prevention in AF (SPAF)] were searched separately with one of the following treatment terms: anticoagulant, antiplatelet, blood thinner or antithrombotic. Other sources searched included FDA and European Medicines Agency (EMA) approved medical product labels for antiplatelet or anticoagulant medications approved through the years 2010 to 2020 for the indications of CAD/ACS, AF, or stroke who require antithrombotic treatment for reducing risk of future thrombotic events; guidance documents (regulatory, clinical, or expert guidance) and scientific recommendations published from 2015 to 2020; and gray literature. In addition, the online Patient-Reported Outcomes and Quality of Life Instruments Database (PROQOLID) of over 2300 clinical outcome assessments (COAs) was reviewed for any PRO instruments for the thrombotic indications.

#### HTA evidence use analysis

A targeted search of data available regarding HTA decisions for antithrombotic medications from major HTA organizations was conducted between 29th September and 15th October 2020, and documents for HTA decisions in cardiovascular-related thrombosis were reviewed to identify any reference to consideration of PRO data and COA measures being used to capture this. The HTA organizations included were: Canadian Agency for Drugs and Technologies in Health (CADTH), Institut National d’Excellence en Santé et en Services Sociaux (INESSS) (Canada), European Network for Health Technology Assessment (EUnetHTA) (EU), Gemeinsamer Bundesausschuss (G-BA) (Germany), Haute Autorité de Santé (HAS) (France), National Institute for Health and Care Excellence (NICE), and Scottish Medicines Consortium (SMC) [United Kingdom (UK)]. Products reviewed included apixaban, cangrelor, dabigatran, edoxaban, prasugrel, rivaroxaban, ticagrelor, vorapaxar, and warfarin. Decision documents for pharmaceutical interventions that have been published from 2010 onwards were included in the targeted literature review to ensure extracted information is applicable to current decision frameworks. Exclusion criteria included (1) articles published prior to 2010; (2) no discussion of relevant endpoints in the document or no new insights reported (e.g., the same data reported as in previous submissions of the same product); (3) generics or biosimilars; (4) appraisals for alternative/behavioral therapies or procedures rather than drug interventions; and (5) clinical guidelines or other “non-HTA” reviews.

#### Review of instrument measurement properties

Following the qualitative literature, PRO instrument, and HTA reviews and development of the high-level conceptual summary, PRO measures were reviewed that assessed core concept(s) depicted in the conceptual summary. An overview of the psychometric or measurement properties of selected measures including, validity, reliability, responsiveness, and meaningful change estimation, was undertaken to identify how well the instrument is measuring the construct(s) of relevance. The evidence was reviewed for the indications ACS, CAD, SPAF, and SSP. These psychometric properties were evaluated in terms of industry standards and with reference to the 2009 FDA PRO Guidance [11] (Appendix A).

## Results

### Qualitative literature review

**Fig. 1:**
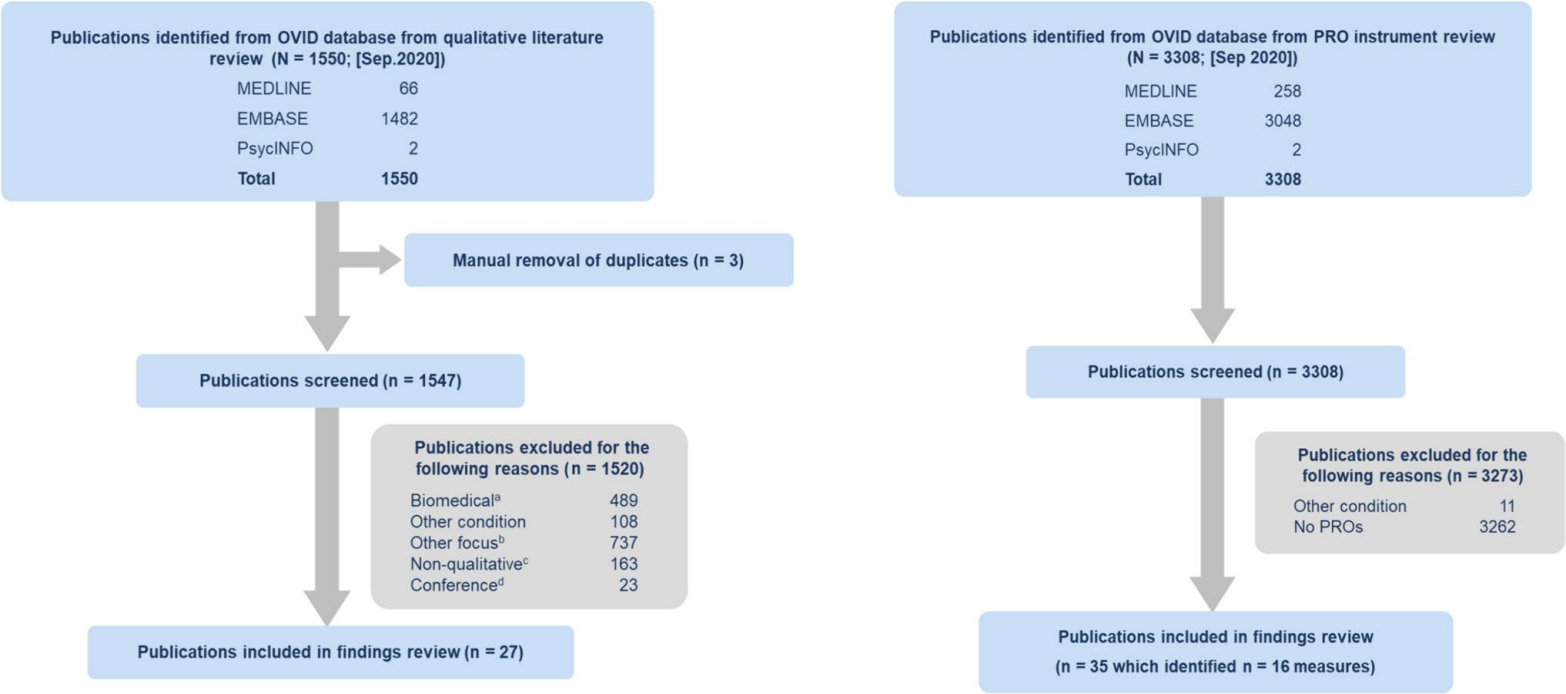
PRISMA diagrams of qualitative literature and instrument landscape review search findings. a Biomedical Exclusion = Studies such as medical devices, procedures, pharmacokinetics, and clinical results of trials with no qualitative patient input. b Other Focus Exclusion = Studies such as usability evaluations, economic evaluations, risk reduction programs, and single-symptom research with variety of indications. c Non-Qualitative Exclusion = Studies such as large-scale surveys without in-depth qualitative interviews. d Conference Exclusion = Conference abstracts with no available manuscripts

The qualitative literature review from the OVID database yielded a total of 1550 publications which resulted in 27 relevant publications (Fig. 1). The main reasons for exclusion were focus on other topics such as usability or economic evaluations and focus on biomedical research such as medical devices.

#### High-level conceptual summary

**Fig. 2:**
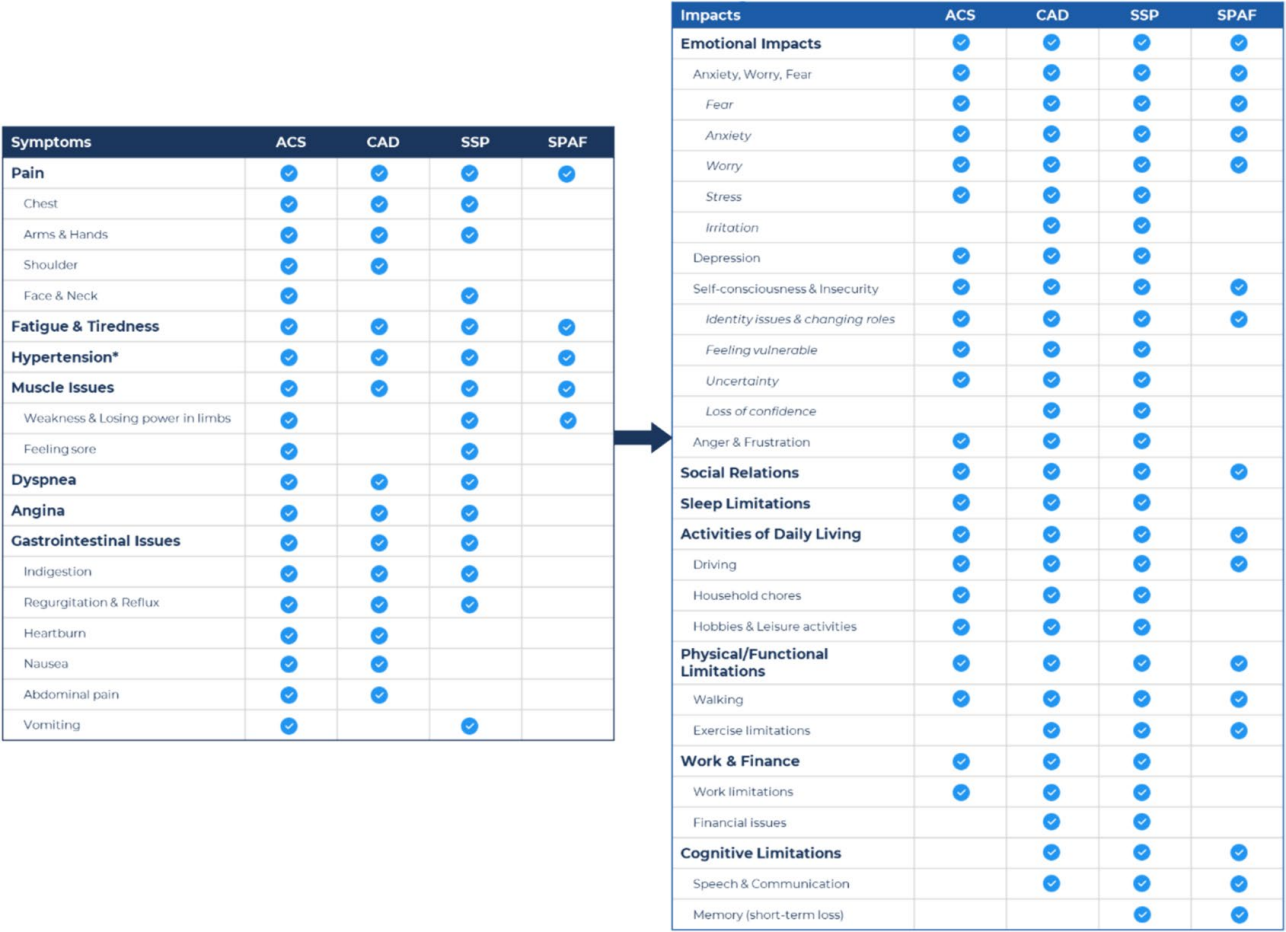
High-level conceptual summary of symptoms and impacts. *Although hypertension is a syndrome and not a symptom, it was reported to be experienced by all patient populations

A high-level conceptual summary depicting the core symptoms and impacts reported to be experienced by patients from at least 3 of the thrombotic indications was developed based on the findings from the qualitative literature review (Fig. 2). Subconcepts reported by patients from at least 2 of the thrombotic indications were also included in the conceptual summary (e.g., shoulder pain subconcept was reported for both CAD and ACS under Pain). Seven core symptoms were identified of which pain, fatigue and tiredness, hypertension, and muscle issues were experienced by patients across all indications. Although hypertension is a syndrome and not a symptom, it was reported to be experienced by all patient populations and was therefore included in the conceptual summary. Seven core impact areas were also identified with emotional impacts, social relations, activities of daily living, and physical/functional limitations being impacts experienced by patients across the indications.

### Instrument review and review of instrument measurement properties

#### Instrument review

Overall, 25 unique PRO instruments were identified through searches of the various sources including OVID publications, clinical trials, PROQOLID, clinical organizations, and HTA documents. The concepts the PROs assessed included mainly HRQoL, treatment satisfaction, and mental health.

The OVID literature review resulted in a total of 16 measures identified from 35 publications (Fig. 1). The measures most used were the EuroQol (EQ)-5D [16] and 36-Item Short Form Survey (SF-36) [17]. The Anti-Clot Treatment Scale (ACTS) [18], which was used in ACS and SPAF, was the measure most identified to capture symptomatic treatment effects associated with anticoagulant and antiplatelet use. The Nuisance Bleeding Scale (NBS) [19], which captures minor bleeding in anticoagulated patients, was also identified. Several other PRO instruments used in similar conditions were also identified.

A total of 16 clinical trials were identified; 13 trials were from ClinicalTrials.gov, and 3 trials were from ClinicalTrialsRegister.eu. Twelve different PRO instruments were identified which were being used as an outcome measure across these trials; 8 of which were unique and not identified in the OVID instrument search. The majority of trials with PROs were those of CAD (n = 10). No measures of symptomatic treatment effects associated with anticoagulant and antiplatelet use such as the ACTS or NBS were identified in any of the trials although, treatment satisfaction [e.g., Treatment Satisfaction Questionnaire for Medication (TSQM)] [20] was measured in one of the trials.

No PROs were included in any of the 9 FDA/EMA medical product labels that were reviewed which included apixaban, clopidogrel, dabigatran, edoxaban, prasugrel, rivaroxaban, ticagrelor, vorapaxar, and warfarin.

No regulatory guidance documents, including FDA and EMA guidance documents, provided any recommendations on PRO instruments to capture symptomatic treatment effects associated with anticoagulant and antiplatelet use or physical function in the thrombotic indications.

Two organizations, International Consortium for Health Outcomes Measurement (ICHOM) and American College of Cardiology (ACC) PRO Forum, provided recommendations on use of PRO instruments in several related conditions and were reviewed. ICHOM has several PRO instrument recommendations for use in CAD, AF, and stroke in general. For CAD, ICHOM recommends use of the Seattle Angina Questionnaire (SAQ-7) [21] to measure angina, Patient Health Questionnaire-2 (PHQ-2) [22] for depression, and Rose Dyspnea Scale for Dyspnea [23–25]. For AF, 12-Item Short Form Survey (SF-12) [26] and Patient-Reported Outcomes Measurement Information System (PROMIS) global health [27] to measure HRQoL and physical and emotional functioning [28, 29]; Atrial Fibrillation Effect on Quality of Life (AFEQT) [30] or Atrial Fibrillation Severity Scale [31] for exercise tolerance and symptom severity; and Work Productivity and Activity Impairment Questionnaire (WPAI General Health v2.0) [32] for ability to work are recommended. For stroke, ICHOM also recommends the PROMIS-10 [33] to measure overall mental wellbeing including cognitive, psychiatric, and social functioning, as well as overall physical wellbeing including pain, fatigue, and general health status [34]. The ACC PRO Forum [35] identified the SAQ (19-item version) [36] as a well-validated PRO measure used in CAD clinical trials [37, 38] and the SAQ-7 (7-item version) [20] for use in clinical practice. Additionally, the AFEQT was identified in AF [39] although further validation to improve the measure is needed, and the Cardiac Self-Efficacy Questionnaire (CSEQ) [40], HeartQoL [41], MacNew Heart Disease Health-Related Quality of Life Questionnaire (MacNew) [42, 43], and SAQ [36] were identified as being developed in patients with coronary heart disease.

The search in PROQOLID resulted in the SAQ for CAD. The indications ACS, SSP, and SPAF provided no results. Following this, 2 separate searches were conducted using the keywords *atrial fibrillation* and *stroke*, which resulted in 5 stroke and 5 AF PRO measures being identified. The stroke measures included Stroke Impact Scale (SIS v2.0) [44], SIS v3.0 [45], SIS-16 [46], Newcastle Stroke-Specific Quality of Life Measure (NEWSQOL) [47], Stroke-Specific Quality of Life Measure (SS-QOL) [48]. The AF measures included Atrial Fibrillation Impact Questionnaire (AF Impact) [49], Atrial Fibrillation Severity Scale (AFSS) [50], Quality of Life Questionnaire for Patients with Atrial Fibrillation (AF-QOL18) [51], AFEQT, and Perception of Anticoagulant Treatment Questionnaire (PACT-Q) [52].

#### HTA evidence use analysis

The targeted HTA website searches identified 27 evaluation documents. A total of 5 different PROs were identified in the search, and all HTA agencies (NICE, SMC, HAS, G-BA, INESSS, CADTH) discussed EQ-5D consistently in their documents. NICE and SMC discussed ACTS and TSQM in the review of rivaroxaban (Xarelto); NICE mentioned the SAQ Angina Frequency and Physical Limitations scale scores and the London School of Hygiene Dyspnoea Questionnaire score in its review of prasugrel (Efient). There was no further discussion of the above-mentioned instruments by NICE and SMC in their recommendations.

Reducing the risk of bleeding was consistently discussed in France (HAS), Germany (G-BA), Canada (CADTH, INESSS), and UK (SMC, NICE) reviews. The UK assessment identified the inability to demonstrate HRQoL loss associated with stroke and major bleed as one of several limitations of the health economic evidence cited for not adequately demonstrating an economic case for the product. The INESSS review discussed concern about some patients with AF not currently receiving anticoagulant therapy due to fear of bleeding (e.g., patient at high risk of falls or having a history of gastrointestinal ulcer as well as alcoholism). The G-BA mentioned that the collection of HRQoL could have been used to provide information as to whether bleeding events impact HRQoL significantly, which might have altered their recommendation for listing the product. Finally, PRO data did not contribute to the overall decision if the efficacy data do not show a significant and relevant improvement.

#### Instrument measurement properties

Following the instrument review, key psychometric properties of 11 selected PROs and levels of psychometric evidence supporting use of each measure in patients with ACS, CAD, and SPAF, and SSP, or a similar indication were evaluated. High-level findings are reported in Table 2. The review revealed evidential gaps in at least 1 psychometric property for all 11 measures evaluated in the thrombotic indications. In particular, evidence of construct validity, central to the measurement of concepts, was found in similar populations but was lacking in the indications of interest, and 6 of the 11 measures lacked evidence of meaningful change in all the indications.

**Table 2:**
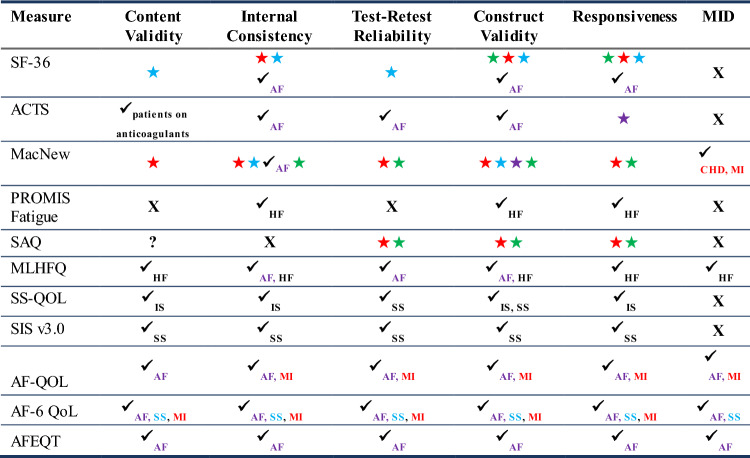
High-level psychometric properties reviewed for PROs (n = 11)

Subscripts by check marks indicate different indications where psychometric evidence were found other than the indications of interest. If a condition is similar to one of the indcations, then they are in the same star color (e.g., MI is considered related to CAD, thus it is in red)

Key: Red star = supporting evidence in CAD; Green star = supporting evidence in ACS; Blue star = supporting evidence in SSP; Violet star = supporting evidence in SPAF; ✓ = supporting evidence at a similar population; ? = questionable evidence; ✕ = no supporting evidence provided

*ACS* acute coronary syndrome, *ACTS* Anti-Clot Treatment Scale, *AF* atrial fibrillation, *AF-6* Atrial Fibrillation-Specific Questionnaire, *AFEQT* Atrial Fibrillation Effect on Quality of Life, *AF-QOL* Quality of Life Questionnaire for Patients with Atrial Fibrillation, *CAD* coronary artery disease, *CHD* coronary heart disease, *HF* heart failure, *IS* ischemic stroke, *MacNew* MacNew Heart Disease Health-Related Quality of Life Questionnaire, *MI* myocardial infarction, *MID* minimally important difference, *MLHFQ* Minnesota Living with Heart Failure Questionnaire, *SAQ* Seattle Angina Questionnaire, *SPAF* stroke prevention in atrial fibrillation, *SS* stroke survivors, *SSP* secondary stroke prevention, *SS-QOL* Stroke-Specific Quality of Life Measure

## Discussion

These types of PRO landscape reviews are often conducted to obtain patient experience data and to identify relevant PRO instruments through a review of different data sources. Ultimately, these data are used to inform a COA strategy for a clinical development program and future research needs to support that strategy. The objectives of this study were met, although with some limitations. For the first study objective, symptoms and impacts relevant to patients with CAD/ACS and receiving treatment for stroke and AF were identified through a qualitative literature review and depicted in a high-level conceptual summary. In the development of this conceptual summary, few qualitative articles (n = 2) on SPAF were found which limited the information on symptoms and impacts. In addition, few qualitative studies (n = 4) mentioned patients were using antithrombotic drugs during data collection, and fewer (n = 2) mentioned treatment effect. Data were largely extracted from a limited number of participant quotes and brief author descriptions within each article. Some of the studies were focused on experiences related to diagnosis, hospitalization, delay in seeking treatment, and major adverse cardiovascular events. Thus, the conceptual summary represents both patient experience of these events as well as long-term adjustment after these events, and while the geographic region of articles was diverse, it was still skewed towards developed countries.

The second objective focused on a review of PRO instruments used in clinical research and practice, to assess symptoms and HRQoL impacts, including those related to physical function and symptomatic treatment effects associated with anticoagulant and antiplatelet use and physical function, in patients with CAD/ACS, AF, and stroke to determine their appropriateness for use in a clinical trial. A total of 25 PRO instruments measuring mainly the concepts of HRQoL, treatment satisfaction, and mental health were identified. Overall, the instrument review highlighted gaps in evidence supporting the psychometric properties of many of these measures and the need for the development of de novo disease-specific, fit-for-purpose measures for use in clinical trials of thrombotic vascular indications or the adaptation of existing measures. The most used measures were the EQ-5D and SF-12, both generic measures of HRQoL. No measures of symptomatic treatment effects related to anticoagulant or antiplatelet use were included in any of the clinical trials. In addition, the FDA/EMA label review yielded no findings of PROs in any of the medical product labels, and guidance on inclusion of PRO instruments in clinical trials of these indications by regulatory agencies such as FDA or EMA or key clinical organizations is lacking or minimal.

A targeted search and review of available data for HTA decisions for thrombotic medications from major HTA organizations, which was conducted to meet the third objective, yielded limited reference to consideration of PRO measures being used to capture symptoms and impacts relevant to the patient populations, including bleeding associated with anticoagulant or antiplatelet use and physical functioning. Findings reveal that PRO data may not be considered in HTA decisions if treatment does not show statistically significant results. Yet, HTA agencies recognize the importance for a new treatment to offer better outcomes, and bleeding risk is taken into consideration along with preventing future cardiovascular events. It is important to note that the HTA review was limited to information available in the public domain, and therefore, the transparency of reporting by each HTA agency. Not all PRO instruments included in the clinical trials were reported in the HTA documents suggesting an element of selection, most likely by the company submitting the evidence or the HTA review committee. Therefore, HTA reviewers are likely only commenting on what they were presented and may only comment if they perceived an issue with the instrument, analysis, or outcome. There were no references to patient or physician valuation of the endpoints in the documents. However, one of the strengths of reviewing the evidence as summarized by the agencies was that we can see which measures they deem to be relevant and whether it influenced the final decision.

Overall, the core symptoms and HRQoL impacts depicted in the conceptual summary can have significant effects on the lives of patients with these conditions. Psychological issues such as anxiety, stress, social isolation, and depression have been reported as risk factors for coronary heart disease [53–56]. Mobility limitations can also increase risk of thrombotic indications [57, 58]. The experience of hypertension and reported symptoms (e.g., headache, dizziness, nausea, and confusion) is important, and effective clinical management of a patient needs to consider overall cardiometabolic health. Further, treatment of thrombotic indications is often lifelong, and an area of clinical concern regarding anticoagulant/antiplatelet therapy is medication non-adherence [59–62]. Poor physical and mental health, fear of nuisance bleeding, depression, and cognitive decline have been implicated as factors in non-adherence [63–65]. Therefore, it is important to ensure that all relevant factors are adequately captured from the patient’s perspective and measured in clinical studies. Yet, findings from the landscape review revealed that there were some challenges to identifying PRO instruments that capture symptoms and HRQoL in patients with CAD/ACS, stroke, and AF, as well as symptomatic treatment effects associated with anticoagulant and antiplatelet use and physical function, to use in clinical studies. Specifically, there is a lack of thrombotic indication-specific PRO instruments. In addition, inclusion of PRO instruments in clinical trials and guidance on inclusion by regulatory agencies or key clinical organizations was lacking or minimal. The emphasis on clinical endpoints in studies of these conditions may also undermine the motivation for including PROs in clinical studies. However, while clinical endpoints are measures of objective clinical efficacy and effectiveness, they do not inform patients’ HRQoL emotional state or attitudes towards treatment interventions; PROs complement clinical endpoints and provide a more comprehensive understanding of treatment outcomes. Consultation with regulatory agencies and optimizing PRO instruments to address thrombotic, disease-specific, HRQoL symptoms and impacts may help mitigate these challenges. The development of core sets of PROs is gaining momentum in various therapeutic areas; the FDA has recently issued a guidance on core PROs for cancer clinical trials. Thus, development of a core set of PROs for these cardiovascular indications may be useful for clinical trials and may also inform healthcare providers of the patient’s HRQoL, leading to improvement in clinical outcomes.

There are a few overall study limitations. The high-level conceptual summary was developed using literature only, which is often a starting point to help guide future research with patients. The conceptual summary should be further evaluated via patient and clinician interviews to help inform the summary, confirm the reported symptoms and impacts, and ensure that none are missing. Inclusion of the patient’s voice into drug development and evaluation is crucial. In terms of the review of instruments within the clinical trial databases, it is important to note that not all trials list all secondary or exploratory outcomes included and therefore the search may not have captured all PROs included in clinical trials. For the OVID COA search, other terms may be considered, such as those relevant to the experience of hypertension or raised blood pressure, which play an important role in cardiovascular conditions, to ensure these are captured by the measure. Other measures may have been identified, although unlikely as general terms for symptoms were included. Further, the HTA review identified information on past decisions and does not reveal what the current policies are and how they may be evolving.

## Conclusion

In this study, a landscape review of several sources was performed to understand symptoms and impacts important to patients with CAD/ACS, stroke, and AF who require antithrombotic treatment for reducing risk of future thrombotic events and to identify relevant PRO instruments. Based on our findings, a high-level conceptual summary was developed encompassing symptoms and HRQoL impacts across these indications. Findings from the PRO instrument review revealed limitations in the psychometric properties of the 11 measures evaluated for use in these indications and the need to develop a core set of disease-specific PRO instruments that can be used in clinical trials and are accepted by regulators and HTA bodies.

## Supplementary Information

Below is the link to the electronic supplementary material.

Supplementary file1 (DOCX 15 KB)

## Data Availability

The data that support the findings of this study may be made available from the corresponding author upon reasonable request, depending on confidentiality level of the requested data.
