# Supplementary material for: A landscape review to identify what matters to patients with thrombotic cardiovascular diseases and patient-reported outcome instruments which can be used to capture the patient experience
Source: Qual Life Res. 2024 Nov 22;34(1):101–11. doi: 10.1007/s11136-024-03790-1 (PMC11802703; doi:10.1007/s11136-024-03790-1)
Supplement: Supplementary file 1 — Supplementary file1 (DOCX 15 KB) [file 11136_2024_3790_MOESM1_ESM.docx]

**Appendix A: Industry Standards used for Evaluation of Psychometric Properties**

Minimum thresholds were provided for floor/ceiling effects, Cronbach’s alpha which assesses the degree of homogeneity among items in a domain (for assessment of internal consistency reliability), intra-class correlation coefficient (ICC) which measures the degree of association between repeated scores on the instrument in stable patients (for assessment of test-retest reliability), correlation coefficients between the measure and other measures known to measure similar or dissimilar constructs (for assessment of convergent/concurrent and divergent validity) and effect size (for assessment of known groups validity and responsiveness). These thresholds are: for floor/ceiling effects >20-25% of the sample with scores in the lowest and highest response categories; 0.70 or 0.80 for Cronbach’s alpha; 0.70 or 0.80 for ICC; small, medium, and large correlations of < 0.4, ≥ 0.4 – < 0.7, and ≥ 0.7, respectively; effect sizes indicating small change/difference (0.20); moderate change/difference (0.50); and large change/difference (0.80). Meaningful change is the level of difference in scores in the domain of interest (eg, symptom or functional score) which patients perceive as meaningful. This may be estimated at the individual patient level as a meaningful change threshold, or at the group level as a minimum important difference.
